# Current Perspectives in Mesenchymal Stem Cell Therapies for Osteoarthritis

**DOI:** 10.1155/2014/194318

**Published:** 2014-12-08

**Authors:** Baldur Kristjánsson, Sittisak Honsawek

**Affiliations:** ^1^Department of Biochemistry, Faculty of Medicine, Chulalongkorn University, King Chulalongkorn Memorial Hospital, Thai Red Cross Society, 1873 Rama IV Road, Pathumwan, Bangkok 10330, Thailand; ^2^Department of Orthopaedics, Faculty of Medicine, Chulalongkorn University, King Chulalongkorn Memorial Hospital, Thai Red Cross Society, 1873 Rama IV Road, Pathumwan, Bangkok 10330, Thailand

## Abstract

Osteoarthritis (OA) is a degenerative joint disease most commonly occurring in the ageing population. It is a slow progressive condition resulting in the destruction of hyaline cartilage followed by pain and reduced activity. Conventional treatments have little effects on the progression of the condition often leaving surgery as the last option. In the last 10 years tissue engineering utilising mesenchymal stem cells has been emerging as an alternative method for treating OA. Mesenchymal stem cells (MSCs) are multipotent progenitor cells found in various tissues, most commonly bone marrow and adipose tissue. MSCs are capable of differentiating into osteocytes, adipocytes, and chondrocytes. Autologous MSCs can be easily harvested and applied in treatment, but allogenic cells can also be employed. The early uses of MSCs focused on the implantations of cell rich matrixes during open surgeries, resulting in the formation of hyaline-like durable cartilage. More recently, the focus has completely shifted towards direct intra-articular injections where a great number of cells are suspended and injected into affected joints. In this review the history and early uses of MSCs in cartilage regeneration are reviewed and different approaches in current trends are explained and evaluated.

## 1. Introduction

With an ageing population and increasing life expectancies, age-associated diseases are becoming a major public health concern. Osteoarthritis (OA) is a destructive joint disease, causing degeneration of cartilage, changes in the subchondral bone and synovium, followed by damage to the underlying bone, and morphological changes such as subchondral sclerosis, subchondral bone cysts, osteophyte formation, and synovitis [1–3]. A number of risk factors have been linked with OA including age, genetic predisposition, hereditary factors, obesity, mechanical injuries, and joint trauma [4, 5]. It most commonly occurs in the elderly and can affect all joints in the human body with weight bearing joints that are frequently under mechanical stress being the major sites [6]. Neuropathic pain, depression, and sleep disorders have also been associated with OA, which further increases its economic burden on society [7]. Even with OA being such a common condition, no approved medical treatment that reverses the destruction of the articular cartilage currently exists [8]. OA is a slowly progressing condition which can go unnoticed for years, the lack of biomarkers and low public awareness have made the early detection of OA challenging. Conventional treatment, for instance, physical therapy, pain control with steroidal, and nonsteroidal anti-inflammatory drugs, and viscosupplementation with injections of hyaluronic acid (HA) can relieve pain, but none of them have an impact on the progression of the condition [9, 10].

Cellular therapies for treating early to late stage OA have also been around for over two decades. Autologous chondrocyte implantation can repair and restore cartilage, but it is a slow process and often leads to insufficient results due to the poor self-renewal and regeneration potentials of chondrocytes [11, 12]. Moreover, it is an invasive method requiring surgery to obtain cartilage from nonweight bearing joints and another surgery to apply them to the affected site. The lack of successful conventional treatments often leads to arthroplasty in end-stage OA patients. Total knee arthroplasty is a surgical procedure wherein the dysfunctional joint surface is replaced with an orthopaedic prosthesis. In recent years, researchers' focus has shifted towards less invasive treatments to regenerate full thickness articular cartilage such as the use of mesenchymal stem cells. A number of case reports and clinical trials have been published showing that mild to moderate OA can be treated efficiently in a simple way using autologous or allogenic mesenchymal stem cells. This review will seek to explain mesenchymal stem cells roles in OA and how they can be recruited for cartilage repair.

## 2. Mesenchymal Stem Cells

Mesenchymal stromal cells or mesenchymal stem cells (MSCs) are multipotent progenitor cells. First described as fibroblast precursors within the bone marrow in 1966, they have since been shown to exhibit vast mesodermal differentiation potentials able to give rise to osteocytes, adipocytes, chondrocytes, myoblasts, and tenocytes [13, 14]. In addition, they are able to differentiate into nerve cells and hepatocytes and can be considered as partly pluripotent [15, 16]. MSCs are involved in the maintenance and regeneration of connective tissues and are known to migrate to tissues as a result of injury or inflammation where they participate in the repair of damage [17, 18]. They are immunoprivileged cells with immunosuppressive and trophic properties by inhibiting the proliferation of CD4+ and CD+8 T-cells, B-cells, and natural killer cells [19]. MSCs are known to secrete a number of cytokines including PGE2, GM-CSF, IL-1RA, IL-7, IL-8, IL-10, and IL-11, chemokines such as SDF-1, and growth factors [20–23].

MSCs are adult stem cells and, unlike embryonic stem cells, MSCs do not show unlimited self-renewal capacity and cannot be maintained and expanded indefinitely* in vitro*. They can be found in numerous tissues; although they reside predominantly within the bone marrow, but other common sources include adipose tissues, skeletal muscles, umbilical cord blood, and Wharton's Jelly [24–26]. Whilst being a well-studied and a widely used cell line bone marrow-derived MSCs (BMSCs) make up only a small fraction, estimated to be only 0.001%, of the mononuclear cells found in the bone marrow [27]. Under normal culture conditions, MSCs display a fibroblast-like morphology, are adherent to plastic, and can form colonies from single cells referred to as colony-forming fibroblast units [23]. They display the surface antigens CD73, CD90, and CD105, while lacking the expression of the haematopoietic antigens CD11b, CD14, CD34, CD45, CD79, CD19, and HLA-DR [28]. In order to standardise the human MSC field, minimum criteria for defining human MSCs have been put forward by the International Society for Cellular Therapy [29]. However, not all MSCs fall under these definitions such as a subpopulation of BMSCs and adipose-derived MSCs (AMSCs) that are nonadherent to plastic but still exhibit all the other properties of MSCs [30, 31]. The properties of MSCs can be used for therapeutic applications to treat a number of conditions such as autoimmune diseases, diabetes mellitus, multiple sclerosis, and osteoarthritis [32–34]. Before MSCs can be used in treatment they are usually expanded* in vitro* first to create sufficient numbers to work with. However, extensive passaging results in loss of function in addition to mutations and possible tumour genetic effects. Transplantations into immunodeficient animals have shown no evidence of tumour formation and recent studies have revealed that, unlike MSCs from many mammals, human MSCs do not undergo spontaneous transformation when cultured* in vivo*. Notwithstanding, they have been shown to support tumour growth by supporting the growth of the tumour stroma, and the risk should not be underestimated [35–37].

## 3. Mesenchymal Stem Cells in Joints

Joints consist of several tissues mainly originating from the mesoderm and unsurprisingly MSCs can be found in both synovial and solid joints as well as the ligaments of the mammalian body. In the human synovial joint they were first described by De Bari et al. when they successfully isolated MSCs from the synovial membrane in 2001 [38]. They have also been isolated and characterised in the meniscus, ligaments, fat pad, and cartilage of the synovial joint suggesting that MSCs play a crucial role in the maintenance and function of these tissues [39–42]. These cells are similar to MSCs from other sources and capable of self-renewal and trilineage differentiation, but MSCs from the synovial fluid have been shown to exhibit greater clonogenicity and chondrogenic capacity than those from bone marrow. Furthermore, they showed clonal heterogeneity with individual clonal populations exhibiting variable proliferation and differentiation potentials [43]. They might therefore seem like an obvious choice for cartilage repair and trials using rabbit models have shown promising results [44]. However, difficulties in extraction and limited studies of synovial-derived MSCs have favoured other sources of cells and heretofore no clinical trials have been performed in humans.

**Figure 1:**
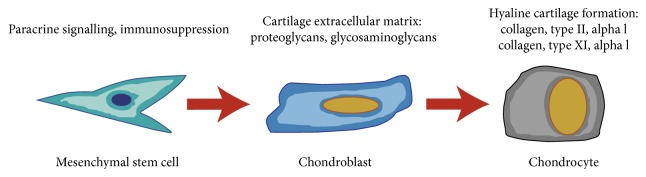
Chondrogenic differentiation and the way in which MSCs can contribute to articular cartilage repair.

Whilst MSCs are widely distributed within the synovial joint, their function has not been fully elucidated. It is likely that they play an important role in providing an opulent reservoir of repairing cells that can be activated for growth, repair, and remodelling. Another function might be to reduce inflammation by suppressing the activity of T-cells [45]. MSCs can be found in cartilage, albeit they seem to lack the ability for functional repair just like chondrocytes, as it is well known that cartilage fails to regenerate following injury. Whereas MSCs are precursors of chondroblasts, which are immature chondrocytes, they might also serve other purposes such as replenishing the surface zone with proteoglycan lubricant to minimise friction within the joint (Figure 1) [46].

## 4. Mesenchymal Stem Cells and Osteoarthritis

Despite MSCs playing an essential role in supplying recovery cells, they also contribute to pathological conditions such as tumour metastasis, aortic valve calcification, and myelofibrosis [47–49]. This might also be true for OA wherein a significantly greater number of MSCs can be recovered from the affected joints of OA or rheumatoid arthritis patients, as well as those of ligament injury, compared with that from healthy joints. Furthermore, the number of MSCs also increases with the severity of the disease suggesting that they might originate in the degrading synovium [50]. In 2002, Murphy et al. showed that MSCs from patients with end-stage OA had reduced* in vitro* proliferation and differentiation potentials. They compared BMSCs from patients who underwent total knee arthroplasty surgery with samples from matched healthy individuals. They observed a significantly reduced yield and proliferation activity along with decreased chondrogenic and adipogenic activity, and increased osteogenic activity [51]. Similar results were produced for retropatellar fat pad-derived MSCs from elderly OA patients showing that age and osteoarthritic condition had significantly reduced the differentiation capacity and expression of stemness genes [52]. More strikingly, it has been evinced that synovial fluid from donors with osteoarthritis or rheumatoid arthritis inhibits the chondrogenic differentiation of MSCs from healthy donors [53]. Factors secreted by the synovial membrane of OA patients also show similar results. It has been observed that these functional deficiencies can be improved with supplementation of the medium with growth factors [54]. Furthermore, the inhibition of protein kinases TAK1 and JAK can reverse this giving MSCs back their chondrogenic potentials, even when grown under OA conditions [55]. Taken together, this suggests that even allogenic cells from healthy donors might struggle to form healthy cartilage that integrates into the host's cartilage under OA conditions, but by adding soluble factors the regeneration could be greatly ameliorated. Notwithstanding, this has not yet been tested* in vivo* nor in any clinical trials.

## 5. Sources of Mesenchymal Stem Cells for Cartilage Repair

A number of factors have to be taken into account when selecting a source of MSCs. Harvesting the cells should result in minimum morbidity to the patients or donors and collection should not result in tissue defects. The source should yield a sufficient amount of functional MSCs that can be expanded easily in culture and introduced to the target site without causing host rejection or further cartilage degeneration. Therefore, obtaining cells for tissue engineering can be a major technical issue. Hitherto, the most common sources of MSCs for treating cartilage damage have been autologous BMSCs, and recently the focus has also shifted towards AMSCs. BMSCs can be collected easily without causing tissue defects by drilling into the bone and aspirating the bone marrow [56]. Major harvest sites include the iliac crest, tibia, and femur, all of which can yield a plethora of bone marrow, from which MSCs can by isolated from and expanded. Adipose tissues are also considered a good source of MSCs and it has been estimated that up to 1,000 times more MSCs can be obtained from each gram of adipose tissue compared to bone marrow making it a very potent source [57]. Use of autologous cells from other sources has also been suggested but has never reached the level of clinical trials in humans, one of the reasons being that in order to obtain cells from these sources more invasive measures have to be applied. Before cultivated cells are applied in treatment they are usually confirmed as MSCs by immunophenotyping; if this is not done, there is no way of knowing if the cells truly are MSCs or simply a niche of unipotent cells that were able to proliferate under the given culture conditions.

MSCs are an advanced medical therapy and as such they should comply with the good manufacturing practice (GMP) guidelines for medicinal products. However, a therapy that utilises living cells cannot be standardised to the same extent as chemically synthesised medicine and the GMP guidelines for medicinal products is not yet fully capable of dealing with cell based therapies. GMP guidelines vary between countries and regions; in USA the Food and Drug Administration (FDA) provides the guidelines, whilst in Europe the European Medicines Agency does. The FDA has defined two categories of human cell products: the “minimally manipulated” category and the “more than minimally manipulated” category in which MSCs fall into [58]. GMP production requires a clearly defined and well-documented manufacturing process, which requires validation at every step. For cell based products that includes routine checks of cell isolations and cultures for any infectious agents, unwanted elements, or cross-contamination of other mononuclear cells, as well as keeping an intense record of the cells origin and donors [59]. Although no lab-produced stem cell therapy has been GMP-approved for commercial production in Europe or USA, a number of clinical trials have been approved and conducted under GMP guidelines.

**Figure 2:**
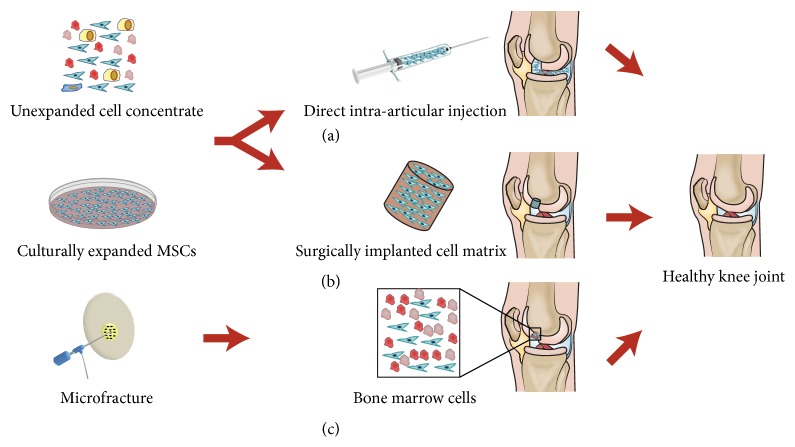
Three different ways in which MSCs can be recruited for articular cartilage repair. (a) Direct intra-articular injections of MSCs in suspensions, (b) surgical implantations of cell sheets or matrixes, and (c) microfracture; drilling into the bone directly recruiting MSCs from the underlying bone marrow.

A number of case reports describe the success of using BMSCs to heal large cartilage lesions (Table 1). The extensive literature on stem cell isolation, chondrogenic differentiation, and composite scaffold design has empowered researchers and clinicians to consider the potential of using stem cells to modify the progression of OA (Figure 2).

## 6. Early Uses of Mesenchymal Stem Cells

The first ever reported use of MSCs to repair cartilage damage in humans was in 1998 by Wakitani et al. [60]. The team successfully transplanted culturally expanded BMSCs (iliac crest) embedded in a collagen gel to full thickness articular cartilage defects in the patellae of a 26-year-old woman. The patient showed significant improvement in pain and walking ability, and arthroscopies 1 and 2 years later revealed that the defects were covered with fibrocartilage. Following this success the team preformed a number of surgeries where they applied this technique with good success.

In 2002 the first report of using BMSCs to treat osteoarthritis was documented by Wakitani et al. in a comparative case control study [61]. The study consisted of 24 patients with knee osteoarthritis who underwent a high tibial osteotomy (HTO). Twelve of these patients received autologous BMSC transplantations and the other 12 served as a control group. BMSCs were introduced in a gel-cell composite which was applied to the abraded areas and covered with collagen sheets. Both groups showed significant improvements in function and muscle strength, in addition to reductions in pain; no difference was observed between the cell-transplanted group and cell-free group. However, during arthroscopy, it was observed that the defects in the cell-transplanted group were covered with white soft tissue and some hyaline cartilage-like tissue. Whilst patients in both groups showed improvement in the quality of life, the additions of BMSCs resulted in the production of cartilage-like tissue* in vivo*.

The pioneering team of Wakitani has reported on a number of case reports where they treated full thickness articular cartilage defects with BMSCs [62, 63]. In 2010 they combined their results and published the first comprehensive study on the safety, effectiveness, and long-term effects of MSC transplantation for cartilage repair [64]. The study was a long-term follow-up study in which they included a total of 41 patients who were operated on. Their results showed that the use of BMSCs was an effective and safe way of treating cartilage defects in most cases. The researchers observed neither tumour formations nor infections during their long follow-up period. Nevertheless, in OA patients, the progression of cartilage destruction could not be reversed in all cases.

## 7. Chondrocytes versus Mesenchymal Stem Cells

Autologous chondrocyte implantation (ACI) can be used to treat symptomatic full thickness articular cartilage or osteochondritis dissecans lesions. Whilst showing excellent results in patients with various cartilage defects that did not respond to previous treatment (excluding OA patients), the results from OA patients have been mixed [65]. Wherefore ACI cannot be considered an adequate treatment for OA patients. Consequently MSCs are being studied as an alternative source of cells for treating cartilage lesions. In a cohort study from 2010, Nejadnik et al. compared the use of GMP produced autologous chondrocytes to GMP produced autologous BMSCs for the treatment of articular cartilage defects [66]. Their study group consisted of 72 matched patients, 36 receiving ACI treatment whereof 15 where suffering from OA and 36 receiving BMSCs treatment of which 20 were suffering from OA. Cell sheets were implanted during ACI surgery where recipients received either chondrocytes or BMSCs. The results showed that patients treated with either ACI or BMSCs had a significant improvement in their quality of life; however, men's health and sport activity showed a greater improvement than that of women. In the chondrocyte group patients older than 45 years had less significant improvements than younger patients, but this was not observed in the BMSC group. This study suggests that both treatments are an effective way of relieving pain and improving the quality of life. The advantages of BMCS treatment are that it requires one fewer surgery and that the surgery is less invasive resulting in lower morbidity and hospitalisation costs. Moreover, treatment with BMSCs showed no difference between age groups. No special remarks were made about the OA patients included in this study.

## 8. “One-Step Repair Technique”

In 2010, Buda et al. introduced a “one-step repair technique” where they used BMSCs to treat twenty patients with osteochondral lesions [67]. Bone marrow aspiration and a standard knee arthroscopy where the cells were delivered were performed in the same operative room without the patient leaving the room. Bone marrow concentrate was embedded in a HA membrane scaffold implanted at the lesion site and covered with autologous platelet-rich fibrin gel to provide growth factors. All patients showed significant improvements in scores measuring pain and OA severity. Magnetic resonance imaging (MRI) revealed that the defect was completely repaired in 14 out of 20 patients. Their approach was simple and resulted in satisfying outcomes for most patients. The whole procedure could be completed in one day and did not require cell expansion or multiple surgeries. Howbeit, it can be assumed that the number of MSCs aspirated and implanted was fairly low compared to cells from expanded cultures. Growth factors might also have played an important role in their success, but the platelet-rich fibrin gel was fabricated from the patient's venous blood. A similar “one-step cartilage repair” was reported by Gobbi et al. in 2011 [68]. Likewise, their patients showed improvements in all scores and the treatment was overall a success confirming that “one-step repair” is an efficient and viable treatment. Additionally, they reported their success applying this technique to 25 patients with symptomatic large chondral defects with good success [69]. The benefits of the “one-step” technique for patients is that it only requires a single surgery where MSCs are both harvested and applied at the same time and it does not require long expansion time or multiple visits to the clinic. The drawback of this approach is that it is hard to estimate the number of MSCs obtained making standard treatment with consistent results more difficult.

## 9. Intra-Articular Injections of Mesenchymal Stem Cells

Major surgical procedures come with high costs and high risks which have led researchers to investigate less invasive methods to recruit MSCs to cartilage lesions. MSCs can be employed in various ways other than just by directly pasting them into the lesion site during open-surgery. The effects of directly injecting MSCs into the knee joints of patients to treat mild to moderate OA has gained interest in the last few years. It was first reported in two case studies by Centeno et al. in 2008 [70, 71]. In their studies they recruited two male patients showing MRI evidence of degenerative knee OA. MSCs were harvested, cultured, and suspended in PBS before being injected into the knee and in the second week the patients received a 1 mL injection of 10 ng/mL dexamethasone, because dexamethasone has been shown to promote chondrogenesis in small doses [23]. Reduction in pain and increased cartilage volume over the 3- and 6-month follow-up times was observed with up to 28.64% cartilage volume increase. These case reports showed that increased cartilage volume and reduction of pain in OA patients could be achieved with minimum invasive measures. However, a major drawback to these case reports was the short-term follow-up; therefore no long-term effects were observed. Following this success, Centeno et al. applied this technique by injecting BMSCs into 227 patients whereof 118 were suffering from knee OA [53]. The main goal of this study was to evaluate the safety of the MSCs injection as well as comparing culturing and delivery methods. No tumour formation was observed at any reimplant site. Furthermore, only three stem cell related complications were reported all of which were minor and easily remedied. Consistent with previous reports [64], this demonstrates the safety of using culturally expanded MSCs in treatment, likewise when using intra-articular injections. In two reports from 2011 and 2012, researchers injected BMSCs into the knees of 10 OA patients [72, 73]. In both studies a slight improvement was observed in few patients for the first 6 months of follow-up but declined in the following 6 months. Taken together, these studies were both promising and encouraging, although not fully satisfactory as a standard treatment for knee osteoarthritis.

More promising results with intra-articular injections of autologous BMSCs alone were produced in 2013 by Orozco et al. [74]. Their study consisted of 12 patients with osteoarthritic knee pain who failed conservative treatment and 9 out of 12 had already undergone previous surgery. Following injections of GMP produced BMSCs pain was significantly reduced at all time points observed. Patients showed rapid and progressive improvement with increased cartilage volume and a lasting pain relief in 11 out of 12 patients. This demonstrated the feasibility and safety of the treatment. It reached up to 78% success with 100% being a perfect treatment. Their results were considerably better than the aforementioned similar studies.

## 10. Postoperative Intra-Articular Injections

In recent years, the effects of postoperatively injecting MSCs or other supporting cells into joints, alone or in combination with assisting agents, have been investigated. Microfracture recruits cells from the bone marrow to cartilage lesions. This is done by drilling small holes into the subchondral bone marrow which stimulates cells from the bone marrow, including MSCs, to migrate to the target site [75]. In a randomised control trial from 2013, Saw et al. investigated the quality of articular cartilage regeneration after arthroscopic microfracture followed by 8 postoperative timely spaced intra-articular injections of HA alone or in combination with autologous peripheral blood progenitor cells (PBPCs) (PBPCs are CD34+, CD105+ cells found within the blood) [76]. The study consisted of 50 patients, 25 of who received PBPCs treatment and 25 serving as a cell-free control group. The intervention group showed significant improvement over the cell-free group in histological and morphological scores. Second-look arthroscopy performed on the majority of patients in both groups confirmed articular cartilage regeneration, and histologic staining suggested the formation of hyaline cartilage, both of which are consistent results with previous findings [77]. This study reveals an alternative cell source for treating full thickness cartilage lesions following a microfracture. The advantages of using PBPCs are that they are relatively easy to harvest in large numbers through apheresis and can be cryopreserved and injected following surgery. Furthermore, they seem to contribute to cartilage regeneration seeing that the intervention group fared better than the control group. Nevertheless, it is hard to determine in what way they contribute to cartilage regeneration as they were injected following microfracture which recruits MSCs from the bone marrow. Whether PBPCs are directly involved in repairing the cartilage and could do it on their own without the help of MSCs, or if they simply serve as supporting cells remains to be seen.

The effects of injecting culturally expanded MSCs following knee surgeries have also been studied. In a report from 2013, Wong et al. compare the effects of postoperatively injecting culturally expanded BMSCs following a microfracture and medial opening-wedge HTO [78]. They enrolled 56 patients with unicompartmental osteoarthritic knees and genu varum in a randomised controlled clinical trial. All patients underwent HTO and microfracture, with the intervention group subsequently receiving intra-articular injections of autologous BMSCs in combination with HA 3 weeks later, whereas the control group received only HA. During the two-year follow-up time both groups showed improvements, but with the intervention group achieving significantly better scores. Furthermore, MRI scans 1 year into the study revealed better cartilage regeneration for the intervention group. In both groups MSCs were introduced by microfracture to stimulate cartilage regeneration. However, it can be concluded that postoperative intra-articular injection of culturally expanded MSCs enhances the effectiveness of the treatment leading to faster and improved cartilage formation. Another advantage of postoperatively injecting MSCs is that they can be harvested during surgery and expanded to adequate amounts before the patient returns to the clinic.

## 11. Adipose-Derived Mesenchymal Stem Cells in Treatment

Although the main focus has been on the use of BMSCs, some researchers have chosen to use AMSCs as an alternative cell line. In 2012 and 2013, Koh et al. published two papers on the same study which revolved around the use of AMSCs for the treatment of osteoarthritis [79, 80]. This study recruited 18 patients who received an injection of AMSCs to the knee. The adipose tissue was harvested from the inner side of the infrapatellar fat pad via a skin incision after arthroscopic debridement. Interestingly, they did not culture the cells but directly isolated them from the fat tissue by centrifuging the tissue sample. Since this was a quick process, they were able to inject the cells back into the patients on the same day as they were harvested. Their data showed a significant reduction of pain and an increased quality of life for all patients and a positive correlation was found between the numbers of cells injected and pain improvements. Furthermore, MRI images taken before and after treatment confirmed that the whole-organ MRI score had increased significantly and the improvement was also correlated with the numbers of cells injected. They concluded that AMSCs were a valid cell source for treating cartilage damage. Their method is also simple and cost-effective with cells being harvested and reinjected into the patient on the same day resulting in reduced costs from cell expansion and from the fact that no hospitalisation is required. The weakness of this study is the same as in the “one-step” technique where the cells were not confirmed as MSCs. Therefore, the cell population might consist of more cell types such as adipocytes. The fact that they observed greater improvements in patients who received higher numbers of cells in their injections is consistent with the antecedent studies [72–74].

As OA most commonly occurs in elderly people it is important to investigate the effects of treatment in that group. In 2013, Koh et al. reported their results on treating 30 elderly OA patients (≥65 years old) who had failed conventional treatment, using intra-articular injections of AMSCs [81]. Patients underwent arthroscopic lavage and cartilage evaluation before receiving an injection of unexpanded AMSCs delivered in platelet-rich plasma (PRP). They demonstrated that AMSC therapy for elderly patients with mild to moderate OA was an effective treatment resulting in reduction of pain and regeneration of cartilage. Facing the same shortcomings as previous studies, the cells were not confirmed as MSCs before injections; therefore the cells injected might not all have been MSCs. Additionally, no quantitative assessment of cartilage regeneration was performed and no control group was enrolled in the study. However, they confirmed leftover cells from treatment as MSCs using immunophenotyping and investigating their differentiation potentials.

## 12. Determination of the Optimal Dose

In 2014 the first dose-dependent study on direct intra-articular injections was published. Jo et al. treated 18 patients suffering from knee OA in a phase I/II clinical trial by autologous AMSC injections [82]. Phase I patients were divided into low dose (1.0 × 10^7^), mid dose (5.0 × 10^7^), and high dose (1.0 × 10^8^) groups, whereas in phase II, 9 patients received the high dose treatment. A significant improvement in joint function and reduction of pain was observed in the low and mid dose groups. The size of the cartilage defect decreased significantly in the mid and high dose groups while increasing in the low dose group. Additionally, arthroscopy and histological staining revealed that a thick hyaline-like cartilage covered the defect sites in the high dose group. The researchers observed no adverse effects and concluded that an injection of 1.0 × 10^8^ cells improved the knees' function by forming cartilage in the defect site. This study bespeaks that larger quantities of cells are more beneficial; it is consistent with other reports where low numbers resulted in poor outcomes. This study also demonstrates the advantage of using AMSCs; they could harvest a great number of cells without expanding them in culture avoiding the cost and time associated with cell culturing. Moreover, it confirms how vital it is to select a good source of cells as they harvested over 100 times more cells from the abdominal subcutaneous fats compared to the fat pads used in earlier studies.

## 13. Allogenic Mesenchymal Stem Cells in Treatment

Whilst a paucity of research exists regarding allogenic MSCs and their potential as a treatment is still being investigated, a milestone was reached in 2014 when Vangsness et al. reported their findings from the first study using allogenic cells [83]. In their in-depth study, they investigated the safety, regenerative effects, and clinical outcomes of intra-articular injections of GMP produced allogenic BMSCs. They recruited 55 patients undergoing partial medial meniscectomy and randomly assigned them into 3 groups. Nonmatched human leukocyte antigen (HLA) allogenic BMSCs from young donors (18–30 years old) where used in the study, making it the first report on the effects of allogenic cells. Groups A and B received 5.0 × 10^7^ or 1.5 × 10^8^ cells while the control group received a cell-free control. During the follow-up they observed no ectopic formations but a number of adverse effects were reported, most of them being mild, such as joint swelling or pain; serious adverse effects were deemed unrelated to the treatment by blinded investigators. An overall significant increase was observed in cartilage volume, although group A, who received 5.0 × 10^7^ showed a better outcome. Significant pain relief throughout the study was observed in both groups A and B while the level of pain remained the same in the control group. Although cartilage increase was not observed in all patients, this study shows that nonmatched HLA allogenic BMSCs can be used in treatment. Due to the destructive nature of OA, this might prove beneficial by using healthy, unaffected MSCs from young donors. Moreover, it eliminates the harvest and cultivation of autologous MSCs, causing less discomfort to the patient and treatment can start contiguously. Another interesting point from this study was that group A, who received a lower number of cells, showed better results in both cartilage volume increase and pain relief. Conversely, in previous studies, higher number of cells produced better results. Albeit the optimal amount of cells has not been determined, group B might have received a plethora when 1.5 × 10^8^ cells were injected.

## 14. Current Clinical Trials

Currently, a number of clinical trials are listed in the National Library of Medicine on the clinicaltrials.gov website. They mainly revolve around the use of expanded autologous MSCs derived from either bone marrow or adipose tissue, although some trials use allogenic or nonculture expanded MSCs. Most researchers focus on the use of intra-articular injections without the use of scaffolds or major surgeries since injections are more cost-effective, cause little morbidity, and are a desirable way of treatment if they are successful. Optimal dosing has not yet been established but in the current trials doses range from 1.0 × 10^7^ to 1.0 × 10^8^. These studies will further help in determining what tissues are good sources of viable MSCs for cartilage repair and what the optimal dose-size should be as well as demonstrating if a single injection is sufficient or multiple injections might be required for satisfying results.

## 15. Conclusion

This review has discussed and evaluated the major ways in which MSCs can be applied in OA treatment through implantation and microfracture as well as intra-articular injections. Findings from the studies described above show that there are alternative means to treat mild to moderate OA. The methods described here have shown promising results but the development of the treatment is ongoing. Better results were obtained with higher numbers of MSCs injected although the optimum dose still remains to be decided. Interestingly, few studies have used multiple injections but instead focused on a single injection hoping it would provide permanent relief of the condition. The results from the single injection studies showed that there was an improvement, but in some cases that improvement was reduced over time. Multiple or even regular injections of MSCs into the joints might be necessary. The ultimate solution would be a single injection of MSCs alone or in combination of growth factors, which would fully regenerate articular cartilage damage and result in a lasting tissue that eliminates the pain which follows the condition. In order to achieve such a dream solution, a number of studies are required with satisfying and consistent results as well as determining all factors of the treatment such as dose-size and vehicles used to deliver and if any external factors are needed. This field merits further investigation.

## Figures and Tables

**Table 1:** Chronological list of publications utilising MSCs for cartilage repair in OA patients.

Study	Study type	Number of patients receiving MSCs	Delivery system	Number of cells	Follow-up time	Control group	MSCs origin	Conclusion
Wakitani et al. 2002 [61]	Case and control study	12	HTO, implantation of cell sheets, gel-cell composite	1.3 × 10^7^	28–95 weeks	12 cell-free controls	Autologous BMSCs from Iliac crest	Autologous BMSCs cell implants are effective for treating OA cartilage defects in humans and producing hyaline-like cartilage

Wakitani et al. 2004 [60]	Case studies	2	Surgery, implantation of collagen cell sheets	N/A	48–69 months	None	Autologous BMSCs from Iliac crest	Autologous BMSCs can be implanted into full thickness articular cartilage defects where they produce fibrocartilage

Kuroda et al. 2007 [62]	Case study	1	Medial parapatellar approach, implantation of collagen cell sheets	5.0 × 10^6^ cells/mL	12 months	None	Autologous BMSCs from Iliac crest	Autologous BMSCs cell implants can promote the repair of large focal articular cartilage defects in young, active patients

Wakitani et al. 2007 [63]	Case studies	3	Medial parapatellar approach, implantation, collagen cell sheets	5.0 × 10^6^ cells/mL	17–27 months	None	Autologous BMSCs from Iliac crest	Autologous BMSCs cell implants can fully cover full thickness articular defects

Centeno et al. 2008 [70]	Case study	1	Intra-articular injection	2.24 × 10^7^	3 months	None	Autologous BMSCs from Iliac crest	Autologous BMSCs can be introduced by intra-articular injections into an osteoarthritic knee which promotes cartilage regeneration and reduction of pain

Centeno et al. 2008 [71]	Case study	1	Intra-articular injection	4.56 × 10^7^	3 months	None	Autologous BMSCs from posterior superior iliac spine	Autologous BMSCs introduced by intra-articular injections into an osteoarthritic knee increase meniscus volume

Nejadnik et al. 2010 [66]	Cohort study	36	ACI surgery, implantation of cell sheets	1.0 × 10^7^–1.5 × 10^7^	24 months	36 patients receiving chondrocyte treatment	Autologous BMSCs from Iliac crest	Autologous BMSCs treatment is as effective as autologous chondrocyte implantations in cartilage repair, additionally reducing morbidity and cost

Buda et al. 2010 [67]	Case series	20	Arthroscopic debridement, implantation, hyaluronic acid membrane scaffold	N/A (2 mL of bone marrow concentrate)	24 months	None	Autologous BMSCs from Iliac crest	One-step repair technique utilising bone marrow concentrate is a simple and time-efficient way to treat large chondral defects

Varma et al. 2010 [84]	Randomized control trial	25	Arthroscopic debridement, intra-articular injection	N/A	N/A	25 cell-free controls	N/A	The technique applied in this study improved the overall osteoarthritis outcome score, especially the quality of life

Centeno et al. 2010 [53]	Case series	227	Intra-articular injection	N/A	Up to 24 months	None	Autologous BMSCs from posterior superior iliac spine	Intra-articular injections of autologous BMSCs are a safe method resulting in no ectopic formations or malignant transformations

Wakitani et al. 2011 [64]	Case series	41	Surgery, implantation, cell sheets, gel-cell composite	5.0 × 10^6^ cells/mL	5–137 months	None	Autologous BMSCs from Iliac crest	Autologous BMSCs implantations are a safe way to treat articular cartilage defects, resulting in no tumour formation or infections

Saw et al. 2011 [77]	Case studies	5	Microfracture, 5 intra-articular injections	N/A (7-8 mL of PBPCs)	10–26 months	None	Autologous PBPCs	Microfracture and injections of autologous PBPCs in combination with HA can regenerate articular hyaline cartilage

Gobbi et al. 2011 [68]	Case series	15	Miniarthrotomy, implantation of collagen cell sheets	Bone marrow concentrate from 60 mL of bone marrow	24–38 months	None	Autologous BMSCs from Iliac crest	One-step repair technique utilising bone marrow concentrate and collagen I/III is efficient in repairing chondral defects

Davatchi et al. 2011 [72]	Case series study	4	Intra-articular injection	8-9 × 10^6^	12 months	None	Autologous BMSCs	Intra-articular injections of BMSCs into OA knees improved the VAS scores; while not excellent, the results were encouraging

Koh and Choi 2012 [79]	Case and control study	25	Arthroscopic debridement, synovectomy, and intra-articular injection	1.2–2.3 × 10^6^	12–18 months	25 cell-free controls	Autologous AMSCs from infrapatellar fat pad	Intra-articular injections of AMSCs are a safe treatment option, reducing pain and improving the function of knee OA patients

Emadedin et al. 2012 [73]	Case series study	6	Intra-articular injection	2.0–2.4 × 10^7^	12 months	None	Autologous BMSCs from Iliac crest	Intra-articular injections of BMSCs into OA knees resulted in no adverse events and improved walking distance and pain scores for the first 6 months, while decreasing in the following 6 months

Orozco et al. 2013 [74]	Case series study	12	Intra-articular injection	40 × 10^6^	12 months	None	Autologous BMSCs from Iliac crest	Intra-articular injections of BMSCs resulted in rapid and progressive improvements, significantly improving quality of life reaching up to 78% improvement

Wong et al. 2013 [78]	Randomized control trial	28	HTO, microfracture, and intra-articular injection	1.46 × 10^7^	24 months	28 cell-free controls	Autologous BMSCs from Iliac crest	Postoperative intra-articular injections of autologous BMSCs improves the MOCART outcomes of patients with varus knees undergoing HTO and microfracture

Koh et al. 2013 [80]	Case series study	18	Arthroscopic debridement, synovectomy, and intra-articular injection	0.3 × 10^6^ to 2.7 × 10^6^	24–26 months	None	Autologous AMSCs from infrapatellar fat pad	Intra-articular injections of AMSCs are a safe treatment option, reducing pain and improving the function of knee OA patients

Koh et al. 2013 [81]	Case series study	30	Arthroscopic lavage, intra-articular injection	4.16 × 10^7^stromal vascular fraction cells (9.7% = 4.04 × 10^6^ stem cells)	24 months	None	Autologous AMSCs from buttocks	Intra-articular injections of AMSCs into OA knees of elderly patients (≥65) are effective in cartilage healing and pain reduction

Saw et al. 2013 [76]	Randomized control trial	25	Microfracture, 8 intra-articular injections	N/A (7-8 mL of PBPCs)	24 months	25 PBPCs free controls	Autologous PBPCs	Microfracture and injections of autologous PBPCs in combination with HA can regenerate articular hyaline cartilage better than microfracture and injection of HA alone

Hauser and Orlofsky 2013 [85]	Case series	6	2–7 intra-articular injections, dextrose prolotherapy	N/A	2–12 months	None	Autologous whole tibial/iliac bone marrow	Intra-articular injections of autologous whole bone marrow are associated with substantial gains in pain relief and functionality

Vangsness et al. 2014 [83]	Randomized, double-blind, controlled study	36	Partial medial meniscectomy, intra-articular injection	5.0 × 10^7^, 1.5 × 10^8^	24 months	20 cell-free controls	Allogenic BMSCs from 18–30-year-old donors	Postoperative intra-articular injections of allogenic BMSCs contribute to meniscus regeneration and reduction in pain following medial meniscectomy

Jo et al. 2014 [82]	Cohort study	18	Intra-articular injection	1.0 × 10^7^, 5.0 × 10^7^, 1.0 × 10^8^	6 months	None	Autologous AMCSs from abdominal subcutaneous fats	Cartilage regeneration and pain reduction are significantly improved when high amounts of AMSCs are injected into OA knees compared with low amounts

Gobbi et al. 2014 [69]	Case series	25	Miniarthrotomy, implantation of collagen cell sheets	Bone marrow concentrate from 60 mL of marrow	Minimum 36 months	None	Autologous BMSCs from Iliac crest	Large chondral defects can be treated with one-step repair technique
